# Rhamnolipid Self‐Aggregation in Water–Bioglycerol Mixtures: Byproduct Valorization for Sustainable Formulation Design

**DOI:** 10.1002/cplu.202500163

**Published:** 2025-06-17

**Authors:** Rodolfo Esposito, Matilde Tancredi, Michela Buonocore, Carlo Carandente Coscia, Francesco Taddeo, Vincenzo Russo, Delia Picone, Gerardino D’Errico, Irene Russo Krauss

**Affiliations:** ^1^ Department of Chemical Sciences University of Naples Federico II Via Cintia 4, Complesso Universitario di Monte Sant’Angelo I‐80126 Naples Italy; ^2^ Consorzio Interuniversitario per lo Sviluppo dei Sistemi a Grande Interfase (CSGI) Via della Lastruccia 3 I‐50019 Florence Italy

**Keywords:** biosurfactants, colloids, crude glycerol, formulations, micellizations, self‐diffusion coefficients, surface tension

## Abstract

Increasing biodiesel production poses a significant challenge in managing its primary byproduct, bioglycerol. Advancements in production techniques have markedly enhanced bioglycerol potential applications across various sectors, including as cosolvent in industrial formulations. In the context of ecosustainable formulation design, this study addresses the self‐aggregation of rhamnolipids, biosurfactants with a wide range of physicochemical and biological activities, in bioglycerol aqueous mixtures, studied by integrating surface tension and NMR self‐diffusion measurements. Preliminary analysis of the properties of water–bioglycerol mixtures indicates that bioglycerol impurities (mainly potassium acetate) have only a small effect on surface tension and no discernible effect on bulk properties such as density, viscosity, and refractive index. The aggregation of rhamnolipids is unaffected by bioglycerol at concentrations up to 40–50 wt%. At higher cosolvent levels, the *cmc* increases and the micellar size decreases, an indirect effect of the decreasing polarity of the medium. These results provide the basic knowledge to promote the exploration of rhamnolipids and bioglycerol as valuable ingredients in formulations for various applications.

## Graphical abstract

Bioglycerol, the primary byproduct of biodiesel production, has the potential to be valorized as a cosolvent in industrial formulations. Surface tension and self‐diffusion data reveal that rhamnolipid self‐aggregation in water is poorly affected by the addition of bioglycerol up to very high concentrations. These results demonstrate that biosurfactants and bioglycerol are suitable ingredients in ecofriendly product design.

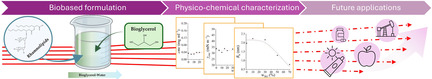

## 1 Introduction

Glycerol is an important byproduct of different industrial processes. In the past, the glycerol obtained from soap manufacturing process, microbial fermentation, or hydrogenolysis of glucose in mixtures of polyols was commonly named bioglycerol.^[^ ^1^ ^]^ In recent years, the term bioglycerol is mostly referred to the principal byproduct of the production of fatty acid methyl esters (FAME),^[^ ^2^ ^]^ by means of the transesterification of rapeseed, soybean, sunflower, or palm oils for the production of modern fuels, such as biodiesel, and fuels components.^[^ ^1^ ^]^

Due to the recent expansion of the biodiesel industries, bioglycerol has gained increasing attention.^[^ ^3^ ^]^ Indeed, in the transesterification process, for each amount of biodiesel produced, it is estimated that 10 wt% amount crude glycerol is produced,^[^ ^4^ ^]^ with potentially detrimental environmental impact.^[^ ^5^, ^6^ ^]^ For this reason, the application of glycerol in industrial products or its conversion into added value compounds is a field of active research.^[^ ^7^ ^]^ There is a wide range of potential uses for bioglycerol, spanning from conversion into added‐value chemicals^[^ ^8^, ^9^, ^10^ ^]^ to the use in bio‐based microbial processes such as enzyme production,^[^ ^11^ ^]^ from the use as raw material for hydrogen and syngas production in the fuel sector^[^ ^12^, ^13^ ^]^ to feedstock in animal feed.^[^ ^14^ ^]^

Bioglycerol from biodiesel industry may contain large amounts of impurities, such as soaps, methanol, FAME, mono‐, di‐, and triglycerides, water, and other organic matter, which depend on the type of oil feedstock used, the efficiency of the overall production process, and on the separation techniques used at the end of the manufacturing process.^[^ ^15^ ^]^ The lowest grade purity bioglycerol (about 50% purity) has very few direct uses, a low commercial value and only marginal application as fuel.^[^ ^16^ ^]^ It represents a possible pollutant for the environment.^[^ ^17^ ^]^ Various methods have been developed to minimize impurities.^[^ ^18^ ^]^ These include development of heterogeneous catalysts used for the conversion of triglycerides to biodiesel, including mixed oxides, basic catalysts, and enzymes, allowing a purity up to 98.5% to be reached without costly downstream purification.^[^ ^19^, ^20^, ^21^, ^22^ ^]^

One of the largest areas of use for bioglycerol, with the potential for further growth, is in the formulation industry. Glycerol finds application in a very wide range of applications:^[^ ^23^ ^]^ it improves the functional properties of formulations by preventing crystallization of the ingredients, influencing rheological properties, affecting distribution and adhesion, and providing the desired smoothness and uniformity.^[^ ^16^ ^]^ Thanks to its nontoxic and highly biocompatible nature, it is often used to increase the viscosity of pharmaceutical and food formulations,^[^ ^23^ ^]^ and as a solvent for different food additives,^[^ ^24^ ^]^ while, thanks to its hygroscopicity, hydrophilicity, and solubility in water, it is used as humectant, i.e., an excipient able to retain water molecules,^[^ ^25^ ^]^ in cosmetic and personal care products,^[^ ^26^, ^27^ ^]^ as well as in household products to prevent drying out,^[^ ^16^ ^]^ and in pesticide formulations to enhance uptake by avoiding droplets evaporation.^[^ ^28^ ^]^

**Figure 1:**
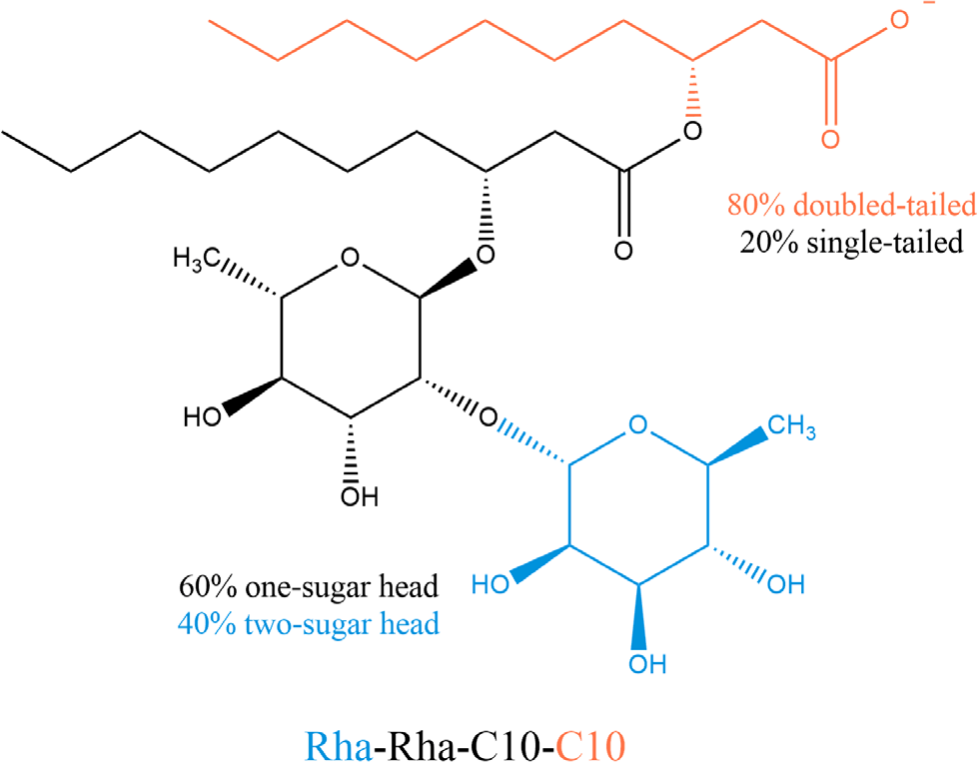
Rhamnolipid molecular structure and composition of the rhamnolipid mixture produced by *P. aeruginosa* used in this work.

Surfactants are key components of most water‐based industrial formulations,^[^ ^28^, ^29^, ^30^, ^31^ ^]^ as they have the peculiar abilities of effectively reducing surface and interfacial tension, solubilizing different classes of additives,^[^ ^32^ ^]^ promoting formation and stabilization of emulsions, and improving foam/gel formation.^[^ ^33^ ^]^ Today, most of the surfactants used in these products are chemically synthesized from nonsustainable and poorly degradable petrochemical resources,^[^ ^34^ ^]^ raising growing concerns for consumer health and the environment.^[^ ^35^ ^]^ Biosurfactants are biodegradable, biocompatible, and sustainable alternatives to synthetic surfactants,^[^ ^36^, ^37^, ^38^ ^]^ being obtained from bacteria, yeasts, or fungi, often using waste materials as carbon sources.^[^ ^39^, ^40^, ^41^ ^]^ Moreover, in addition to surface activity,^[^ ^42^, ^43^ ^]^ they present interesting biological activities, including antimicrobial,^[^ ^38^, ^39^ ^]^ anticancer,^[^ ^44^, ^45^ ^]^ and wound healing.^[^ ^46^, ^47^, ^48^ ^]^ An additional potential benefit is the antioxidant activity, which is being increasingly claimed, but which is still in need of further research for confirmation and understanding.^[^ ^49^ ^]^ The potential application of biosurfactants in chemical formulations aligns very well with the industry's growing focus on natural and sustainable ingredients.^[^ ^50^, ^51^ ^]^ Among biosurfactants, rhamnolipids are particularly promising. They are glycolipids composed of one or two rhamnose units acetylated with one or two long‐chain 3‐hydroxy fatty acids (**Figure** **1**) and obtained as complex mixtures of congeners by *Pseudomonas aeruginosa* and other bacteria. Rhamnolipids strongly reduce surface tension of aqueous solutions in a wide range of pH, salinity, and temperature conditions. Last, they are cheaper than other biosurfactants^[^ ^52^ ^]^ and are effectively obtained from a wide range of waste materials^[^ ^53^, ^54^ ^]^ as the carbon source. Given these advantageous properties and specific biological activities, it has been proposed that rhamnolipids can be used in a variety of applications.^[^ ^55^, ^56^, ^57^, ^58^ ^]^

With the aim of providing the basis for the rational design of green ecosustainable formulations,^[^ ^59^ ^]^ in the present work, we analyze the behavior of a polydisperse rhamnolipids batch, as obtained from biotechnological production, in bioglycerol aqueous solutions. Bioglycerol was obtained as a byproduct of an optimized process for FAME production,^[^ ^60^ ^]^ and subsequently purified.^[^ ^5^, ^15^ ^]^ Preliminarily, the physicochemical features of water–bioglycerol binary mixtures were examined and compared with those reported for pure glycerol. Our analysis focused on density and viscosity, as these parameters not only influence formulation properties but also impact mass and heat transfer within the solution, as well as the feasibility of industrial processes. Additionally, we assessed the refractive index, which affects the visual properties of the formulations, and surface tension, which influences the wetting characteristics. Regarding rhamnolipids, we investigated their aggregation behavior in bioglycerol aqueous mixtures by determining the critical micelle concentration (*cmc*) and evaluated their surface properties in terms of maximum surface tension reduction (*γ*_cmc_) and molecular surface area at the air/liquid interface (*A*_min_). This was achieved by conducting surface tension measurements by changing both rhamnolipid concentration and bioglycerol content. Finally, the intermolecular interactions and the dimension of the rhamnolipid aggregates in the system were investigated by determining the self‐diffusion coefficients of all mixture components using DOSY‐NMR experiments.

## 2 Results

### 2.1 Physicochemical Characterization of Bioglycerol Aqueous Mixtures

In the initial phase of this study, we conducted a physicochemical characterization of the water–bioglycerol binary system, analyzed the results by fitting the experimental data to well‐established models, and compared results with those reported in the literature for water–glycerol mixtures, in order to emphasize any variations that may stem from impurities present in the bioglycerol sample.

**Figure 2:**
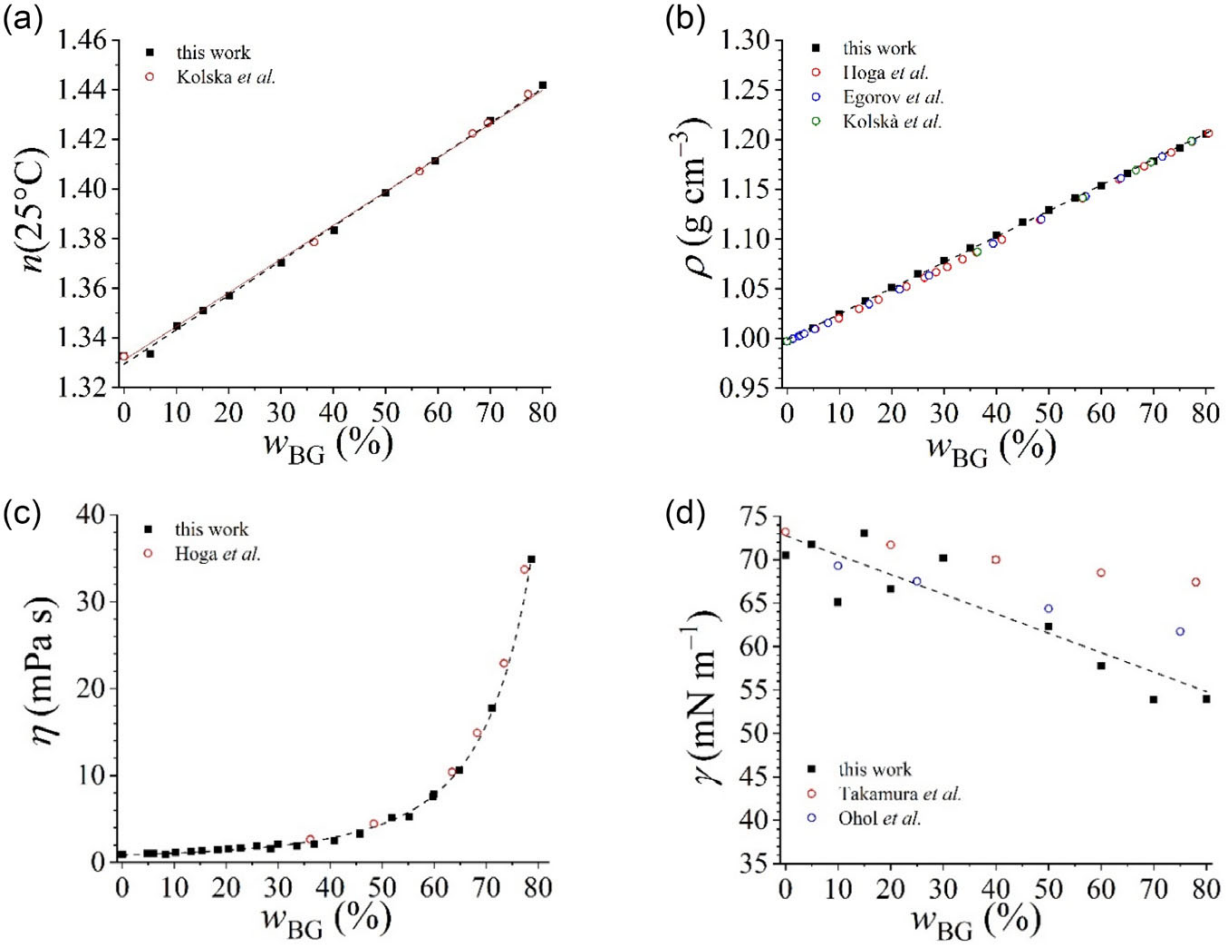
Physicochemical properties of bioglycerol mixtures in 4 mM sodium phosphate buffer solution at pH 7.1, reported as a function of bioglycerol content $w_{\text{BG}}$. **a**) Refractive index, **b**) density, **c**) viscosity, **d**) surface tension. Data in the literature for water–glycerol mixtures are reported for comparison. Best‐fitting curves are also shown as dashed lines.

We first focused on the refractive index of the mixtures at a temperature of 25 °C, *n*(25 °C), given that this feature is particularly responsive to the presence of impurities, and it is a crucial parameter for appearance of formulations, in particular in the cosmetic field.^[^ ^61^, ^62^ ^]^ The refractive index linearly increases with the concentration of bioglycerol (**Figure** **2**a), and the experimental points very well agree with the following linear equation, achieving an *R*‐squared value of 0.998.

$$n(25\;{}^{\circ}C)=(1.3294\pm 0.0008)+(1.39\pm 0.02)\times 10^{-3}w_{\mathrm{BG}}$$

Moreover, the data align closely with previously published results concerning analogous glycerol mixtures,^[^ ^63^ ^]^ demonstrating no discrepancies even at elevated concentrations of bioglycerol (Figure 2a). The refractive index of glycerol–water mixtures can be calculated by summing up the fractional contributions of the pure components ($n(25\;{}^{\circ}C)=n_{H_{2}O}\times w_{H_{2}O}+n_{\mathrm{BG}}\times w_{\mathrm{BG}}=1.333\;\times w_{H_{2}O}+1.474\times w_{\mathrm{BG}}$). Our experimental data were systematically lower than calculated values (Figure S1, Supporting Information panel a), but with deviations less than 0.5%, similarly to findings by Takamura *et al.* ^[^ ^64^ ^]^

Given the role of glycerol as an additive to tune formulation rheological properties, we also determined density and viscosity of the water–bioglycerol mixtures.

The density (*ρ*) of mixtures of buffered phosphate aqueous solutions at pH = 7.1 and bioglycerol linearly increases as a function of bioglycerol content (Figure 2b), in very good agreement with data reported in the literature for aqueous mixtures of glycerol at the same temperature.^[^ ^63^, ^65^, ^66^ ^]^ The data satisfactorily fit to the linear equation (*R‐square* = 0.999)

$$\rho /g\;cm^{-3}=(0.9987\pm 0.0005)+(2.600\pm 0.009)\times 10^{-3}w_{\mathrm{BG}}$$

Also the density of glycerol–water mixtures can be calculated by summing up the fractional contributions of the pure components as done for the refractive index ($\rho =\rho_{H_{2}O}\times w_{H_{2}O}+\rho_{\mathrm{BG}}\times w_{\mathrm{BG}}=0.997\times w_{H_{2}O}+1.252\times w_{\mathrm{BG}}$). We calculated the density of water–glycerol mixtures in this way and found that experimental data were systematically higher than calculated values (Figure S1, Supporting Information panel b), above a 25% bioglycerol content. However, deviations between experimental and calculated values were less than 0.34%. This well agrees with literature results reporting for water–glycerol mixtures at 25 °C a maximum excess volume of −0.35 cm^3^ mol^−1^ at a weight fraction of glycerol of about 70%.^[^ ^62^ ^]^ Considering that the partial molar volume of glycerol in water mixtures at this temperature and composition is about 72 cm^3^ mol^−1^,^[^ ^66^ ^]^ the deviation with respect to the assumption of ideal mixing is less than 0.5%. Therefore, it can be inferred that also water and bioglycerol mix without significant change in their partial molar volumes and the change of volume upon mixing is negligible for most purposes,^[^ ^64^ ^]^ in particular for our purpose of comparing the behavior of bioglycerol and pure glycerol.

The viscosity (*η*) of the aqueous buffered solutions also increases monotonically with the addition of bioglycerol, but it is a highly nonlinear function of the weight percentage of glycerol, reaching values as high as about 34 mPa s at *w*_BG_ ≈ 80% (Figure 2c). The data were well fitted by the Shankar and Kumar equation^[^ ^67^ ^]^ (*R‐square* = 0.998) in the form

$$\eta /mPa\;s=(877.6)exp\left(\frac{\left(0.0259)\times (100-w_{\text{BG}})+(0.00115)\times (0.00371)\times w_{\text{BG}}\times (100-w_{\text{BG}}\right)}{\left(0.00115)\times w_{\text{BG}}+(0.00371)\times (100-w_{\text{BG}}\right)}\right)$$

Experimental data very well agree with results obtained by Hoga *et al.* with aqueous mixtures of glycerol^[^ ^61^ ^]^ up to the highest bioglycerol concentration examined. Figure S2, Supporting Information, shows the same viscosity data as a function of volume fraction, as is often found for liquid binary mixtures.^[^ ^68^, ^69^ ^]^

Finally, we assessed the surface tension of the binary mixtures, as it determines spreading and wetting features of formulations. The surface tension of the aqueous buffered solutions decreases close‐to‐linearly with the weight percentage of bioglycerol, as shown in Figure 2d. The data, although quite scattered at low w_BG_, were acceptably fitted with the following linear expression (*R‐square* = 0.76)

$$\gamma /mN\;m^{-1}=(73\pm 2)-(0.22\pm 0.04)\times w_{\mathrm{BG}}$$

A linear decrease of surface tension with glycerol content was observed also by Takamura *et al.* ^[^ ^64^ ^]^ even if the decrease they found at 20 °C in pure water was much less steep than the one we found, and by Ohol *et al.* ^[^ ^70^ ^]^ whose data more closely agree with our results. In all the cases, a great variability of surface tension values is observed (Figure 2d), and it is worth to note that for mixtures at high content of glycerol, we determined lower surface tension values than those reported for pure glycerol (i.e. 60.54–63.05 mN m^−1^ when the temperature is in the 20–60 °C range).^[^ ^71^ ^]^ This finding could be related to the residual presence of low concentrations of surface active impurities in the bioglycerol sample, which are uneffective in modifying bulk properties such as viscosity, density, and refractive index, but significant for the mixture behavior at the air/water interface.

### 2.2 Aggregation Behavior of Rhamnolipids in Water–Bioglycerol Mixtures

With the aim at analyzing the surfactant effectiveness in the presence of bioglycerol, utilized as a cosolvent, we determined the critical micelle concentration (*cmc*), which is the saturation concentration, beyond which the additional surfactant molecules aggregate to form micelles, and the surface tension at cmc (*γ*_cmc_), as an index of the surfactant's effectiveness in occupying the air–water interface, through a series of tensiometric titrations. In these experiments, the concentration of rhamnolipids (Rha) was systematically increased within buffered phosphate aqueous solutions at a pH of 7.1, while maintaining a constant concentration of bioglycerol, varying from $w_{\text{BG}}$ = 5% to $w_{\text{BG}}$ = 80%. The surface tension of Rha in buffered phosphate aqueous solutions at a pH of 7.1, without bioglycerol, was also assessed for comparative purposes. There was no indication of coacervation or precipitation in any of the surfactant mixtures.

In Figure S3, Supporting Information, plots of the *γ* values as a function of the Rha concentration at the different bioglycerol contents are reported. All the profiles present the typical features expected for surfactant aqueous mixtures, namely a marked inflection point, corresponding to the *cmc*, and a mostly constant surface tension in the micellar range, corresponding to *γ*_cmc_. Up to w_BG_ = 50%, no strong differences due to the presence of the cosolvent are observed, while at higher bioglycerol contents, we found a decrease of the initial surface tension, a certain constancy of surface tension before it starts to decrease at high Rha concentrations, and a shift of the *cmc* toward higher Rha concentrations. On the other hand, *γ*_cmc_ seems to be independent of the bioglycerol content.

Aggregation and surface behavior of Rha in the absence of bioglycerol well agrees with that of the same rhamnolipid mixture,^[^ ^59^, ^72^ ^]^ as well as of purified mono‐ and di‐rhamnolipids in solutions at pH > pK_a_,^[^ ^48^ ^]^ with *cmc* = 0.0606 mg ml^−1^, *γ*_cmc_ = 33.7 mN *m* ^−1^, and *γ*_cmc_ = 67 Å^2^.

**Figure 3:**
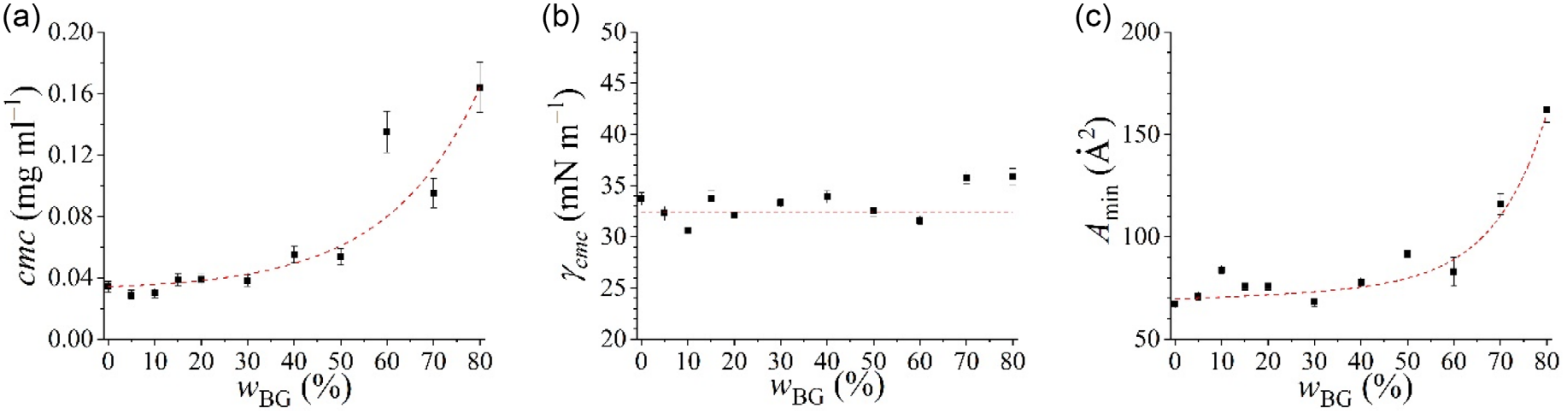
Aggregation and surface properties of rhamnolipids in 4 mM sodium phosphate buffer solution at pH 7.1 as a function of bioglycerol content: **a**) critical micelle concentration (*cmc*), **b**) minimal surface tension (*γ*_cmc_), **c**) area occupied by a surfactant molecule at the air/solution interface (*A*_min_). Red dashed lines are a guide to the eye.

The *cmc*, *γ*_cmc_, and *A*_min_ values are reported in **Figure** **3** (panels a, b, c, respectively) as a function of bioglycerol content to emphasize the effect of the cosolvent. Inspection of Figure 3 highlights that bioglycerol determines a significant increase of the *cmc* (panel a) and the area occupied by a Rha molecule at the air/liquid interface (panel c) at *w*_BG_ > 50%, while the reduction of surface tension induced by Rha is barely affected by the presence of bioglycerol in the whole investigated range (panel b).

The last finding indicates that the cosolvent scarcely contributes to the biosurfactant monolayer at saturation within the range of bioglycerol concentration investigated. On the other hand, the increase of the *cmc* and of the area occupied by a rhamnolipid molecule at the interface at high bioglycerol contents can be both justified by taking into account how glycerol affects the bulk properties of the water solution. In particular, the addition of bioglycerol, characterized by a lower dielectric constant than water, results in a higher electrostatic repulsion between the ionic rhamnolipid headgroups^[^ ^73^, ^74^, ^75^ ^]^ and in a weaker hydrophobic interaction among tails.^[^ ^76^ ^]^ Both factors disfavor the adsorption of Rha molecules at the air–liquid interface as well as their micellization, determining an increase of the *A*_min_ and *cmc* values at high glycerol concentrations.

**Figure 4:**
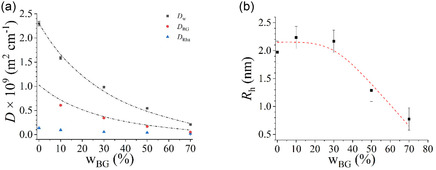
**a)** Self‐diffusion coefficients of water (*D*_w_), bioglycerol (*D*_BG_), and rhamnolipids (*D*_Rha_) in water/bioglycerol/rhamnolipid mixtures at Rha concentration 13.3 mg ml^−1^, as obtained by DOSY NMR experiments, plotted as a function of the bioglycerol content. Error bars, when not visible, are within the data marks. Literature reference trends for water and glycerol are also reported as black and red dash–dot lines, respectively. **b)** Hydrodynamic radius *R*_h_ of Rha aggregates as a function of the bioglycerol content. The red dashed line is a guide to the eye.

To further investigate the self‐aggregation of Rha in aqueous bioglycerol mixtures, a series of DOSY NMR experiments were performed on samples with increasing glycerol content (from *w*_BG_ = 0 to *w*_BG_ = 70 %) at constant biosurfactant concentration (13.3 mg ml^−1^). This technique allows the separate determination of the self‐diffusion coefficients of all the components of the mixture and thus the monitoring of the behavior of each of them. As can be seen from **Figure** **4**a, with increasing *w*_BG_, all of the *D*_w_, *D*_BG_, and *D*_Rha_ values decrease, an evidence that can be coarsely interpreted as due to the increasing mixture viscosity, which also affects the shape and the chemical shift of the DOH signal (Figure S4, Supporting Information). In the same figure, the *D*_w_ and *D*_BG_ trends found in the absence of Rha are also shown.^[^ ^77^ ^]^ It is evident that the mobility of water and bioglycerol is not affected by the presence of the biosurfactant, suggesting no specific/direct interaction to occur between the biosurfactants and the solvent/cosolvent molecules.^[^ ^73^ ^]^

Given that the Rha concentration is considerably higher than the CMC, the monomer contribution is negligible, and the experimental *D*_Rha_ values correspond to those of the micellar aggregates. The Stokes–Einstein equation can be employed to determine the hydrodynamic dimension of these aggregates:

$$R_{h}=\frac{k_{B}T}{6\pi \eta D_{Rha}}$$

In this equation, *k*_B_ is the Boltzmann constant and *η* is the dynamic viscosity of the medium in which the aggregate diffuse. It is noteworthy that this equation strictly holds for noninteracting hard spherical particles diffusing in a continuous fluid. Corrections are reported to account for deviations from both particle sphericity and medium continuity,^[^ ^78^ ^]^ but their application requires information not available in the present case. As a further approximation, when using the Stokes–Einstein equation to calculate the dimension of the Rha aggregates, the dynamic viscosity of the glycerol–water mixtures was used, neglecting the effects that the presence of the solute (the biosurfactant), even at low concentrations, could have on the surrounding medium.^[^ ^79^ ^]^ The low rhamnolipid concentration also makes the obstruction effect negligible.^[^ ^80^ ^]^ Therefore, the computed hydrodynamic radius, *R*_h_, values must be regarded as a qualitative indication of the aggregates’ hydrodynamic dimension, rather than as true geometrical parameters. As illustrated in Figure 4b, *R*_h_ remains almost constant with increasing bioglycerol content, a significant decrease being observed only at *w*_BG_ values greater than 50%. Within the aforementioned approximations, this evidence suggests a reduction of the Rha aggregation number.

## 3 Discussion

The aim of the present study was to strengthen the basis for the use of bioglycerol as a cosolvent in industrial surfactant formulations.

In the first part of the present study, the bioglycerol was widely characterized, focusing on physicochemical properties that affect formulation processability, appearance, and functionality, such as density, viscosity, refractive index, and surface tension. The need for this part of the study arises from the fact that even small amounts of impurities can dramatically affect the properties of a solvent–cosolvent mixture, often in unpredictable directions. The results presented in the Section 2.1 show that the bioglycerol used in this work has bulk properties which do not differ from those reported in the literature for analytical grade purified glycerol in the concentration range up to *w*_BG_ = 80%. This is particularly significant in the case of refractive index because this property is highly sensitive to the presence of impurities in an unspecific manner,^[^ ^81^ ^]^ as some of us have recently shown to occur in the case of binary mixtures of water and bioethanol at high bioethanol contents.^[^ ^59^ ^]^ A fine control of refractive index is crucially used in formulation technology to obtain clear emulsions,^[^ ^82^ ^]^ through the so‐called refractive index matching.^[^ ^83^ ^]^ This approach consists in using an oil phase and a aqueous phase that have equal refractive index values, so that light does not bend as it strikes obliquely at the emulsion interface, but is transmitted through the emulsion without refraction, resulting in a transparent liquid.^[^ ^83^ ^]^ Unpredictable variation in the refractive index of the aqueous phase due to bioglycerol impurities would have prevented its use in this type of formulations.

The only physicochemical property of water–bioglycerol mixtures that differ from literature values is surface tension: in particular, an almost linear decrease of surface tension with glycerol content was observed, as already described in the literature,^[^ ^64^, ^70^ ^]^ but for mixtures at high content of bioglycerol, a lower surface tension value than those reported for pure glycerol^[^ ^71^ ^]^ was found, possibly due to the presence of surface active impurities, which are ineffective in modifying bulk properties, but significant for the mixture behavior at the air/water interface. This interpretation could be supported by evidence reported in the literature that acetate salts, which are the main impurity of the bioglycerol used in this study, cause a decrease in the surface tension of aqueous mixtures.^[^ ^84^ ^]^

A further valuable aspect of this section of the study is that the examination of the data also furnishes an opportunity for a review of the current comprehension of the effect of glycerol on the aqueous medium. According to calorimetric and osmotic measurements,^[^ ^85^, ^86^ ^]^ glycerol is considered to be a structure maker that increases the number of H‐bonds in bulk water. However, by analyzing the trends in transport properties, some of us have found that the water‐structuring effect of glycerol is extremely weak.^[^ ^77^ ^]^ This has been confirmed by a recent spectroscopic study, which shows minimal changes in hydrogen bonding over a wide range of compositions, with an increase detected only at intermediate glycerol contents,^[^ ^87^ ^]^ and by high‐resolution neutron diffraction measurements, indicating that the local structure of water is relatively unperturbed by the presence of glycerol^[^ ^88^ ^]^ and vice versa.^[^ ^89^ ^]^ The density data presented in this study fit this scenario perfectly, showing additivity of component volumes as expected in the absence of significant microstructural changes upon mixing. This is a salient point for the interpretation of the data presented in the following part of the work.

In the second part of this study, the aggregation properties of rhamnolipids (Rha) in the presence of increasing contents of bioglycerol were investigated. In the rhamnolipid molecular structure, rhamnose units act as nonionic polar head, and the carboxyl group acts as an additional anionic head.^[^ ^40^ ^]^ In previous research, it was determined that the rhamnose moieties shield the negative charge of the carboxyl group from the external medium, and the rhamnolipids tend to behave as nonionic surfactants.^[^ ^72^ ^]^

The results presented in Section 2.2 show that up to *w*_BG_ = 40–50% the *cmc* is hardly affected, as is the micellar dimension. Beyond this threshold, the *cmc* increases significantly reaching a value 5 times higher than that in the absence of bioglycerol at *w*_BG_ = 80%, while the size of the micellar aggregates decreases, with the hydrodynamic radius reduced to almost a third of its initial value. In order to comprehend these findings, it is beneficial to summarize the consolidated knowledge on the effect of glycerol on the micellization of the different surfactant classes. A number of researchers have examined nonionic ethoxylated surfactants, determining that glycerol elicits a substantial *cmc* increase, starting from low glycerol content and progressively enhancing, nearly reaching a 10‐fold increase at high glycerol contents;^[^ ^73^, ^90^, ^91^ ^]^ at the same time, the surfactant aggregation number is greatly reduced (up to 50% reduction compared to the value in water).^[^ ^73^ ^]^ Other studies have focused on the behavior of cationic surfactants, especially alkyltrimethylammonium bromides. For these surfactants, a small but detectable increase in *cmc* has been reported at low glycerol content.^[^ ^75^, ^92^ ^]^ The *cmc* increase becomes more pronounced above about 30 wt% glycerol and reaches a threefold increase at 70 wt%.^[^ ^73^, ^93^, ^94^ ^]^ Glycerol also causes a decrease of up to about 40% in the aggregation number^[^ ^73^, ^94^ ^]^ and up to about 20% in the hydrodynamic radius.^[^ ^75^ ^]^ Last, some authors have analyzed the effect of glycerol on anionic surfactants and found little variation in *cmc* at low glycerol contents, a significant increase being observed only above about 30 wt%, to an extent similar to that observed for cationic surfactants.^[^ ^95^, ^96^, ^97^ ^]^ Glycerol has been demonstrated to induce a reduction in aggregation number of up to 30%, accompanied by a decrease of ≈10% in hydrodynamic radius.^[^ ^97^ ^]^ In summary, qualitatively similar effects were observed for all surfactant classes, but the extent of the observed changes varied.

The data presented in this study demonstrate that the alterations in Rha *cmc* in response to bioglycerol are significantly less pronounced than those observed for nonionic ethoxylated surfactants. For these surfactants, the primary cause of the observed increase in *cmc* has been proposed to be dehydration and consequent shrinkage of the bulky headgroup,^[^ ^73^, ^90^, ^98^ ^]^ an effect either absent or only marginally active in the case of Rha. Conversely, the behavior of Rha in bioglycerol aqueous mixtures closely resembles that observed for ionic surfactants. For these surfactants, the observed *cmc* increase is generally interpreted as due to the lowering of the cohesive energy and dielectric constant of the aqueous medium due to the presence of glycerol as cosolvent.^[^ ^71^, ^73^ ^]^ It should be noted that the dielectric constant is a macroscopic property of the medium, while the cohesive energy density is a microscopic concept, being defined as the energy of intermolecular interactions per unit volume.^[^ ^99^ ^]^ Thus, they are strictly correlated^[^ ^100^ ^]^ and, generally speaking, reflect the polarity of the medium.^[^ ^101^ ^]^ As the glycerol content is increased, the cohesive energy and dielectric constant decrease mildly,^[^ ^102^ ^]^ reducing the solvent polarity. This causes the solubility of the hydrophobic tails to increase and the electrostatic repulsion between the headgroups to increase as well, causing a moderate *cmc* increase. An additional consequence of the lowering of medium polarity and increase of electrostatic repulsion is the decrease of dimension of the aggregates: the increased repulsion among the charged headgroups causes an increase of the micellar surface curvature, leading to the formation of smaller aggregates with lower aggregation numbers. Thus, the experimental evidence reported in this work suggests that bioglycerol could enhance the electrostatic repulsion between the Rha carboxylic groups, which becomes more effective than found in water. Based on what was reported in the first part of this discussion, the interpretation of the effect of (bio)glycerol on surfactant micellization in terms of glycerol structure‐making ability on the aqueous medium, which is also proposed in the literature,^[^ ^95^ ^]^ is a minefield.

Data interpretation discussed earlier is supported by the evidence that the area occupied by a rhamnolipid molecule at the interface increases with the bioglycerol content, beyond *w*_BG_ = 40%. The increase in *A*_min_ is unlikely to be ascribable to the coadsorption of Rha and bioglycerol, but rather this finding could be related to the decreased medium dielectric constant and, as a consequence, to the increased electrostatic repulsion among charged Rha molecules at the interface^[^ ^74^, ^75^, ^95^ ^]^ and to the weaker hydrophobic interaction among tails.^[^ ^76^ ^]^ However, this looser packing does not significantly alter the Rha capacity to lower the surface tension. Indeed, *γ*_cmc_ values are mostly unaltered up to *w*_BG_ = 80%, similarly to what found in the presence of the more surface‐active ethanol.^[^ ^59^ ^]^ This suggests a peculiar ability of Rha to displace cosolvent molecules adsorbed at the interface, contrasting with what reported for both anionic^[^ ^71^ ^]^ and cationic^[^ ^92^ ^]^ surfactants for which, with increasing concentration of glycerol, *γ*_cmc_ increases.

## 4 Conclusions

The increasing availability of bioglycerol, the main byproduct of biodiesel production, is encouraging the definition of new applications that are ecologically sustainable, technologically feasible, and economically affordable. In this work, high‐purity bioglycerol, obtained by an optimized FAME process and purification procedure, is used as cosolvent in aqueous surfactant solutions. A preliminary physicochemical characterization of water–bioglycerol mixtures showed no deviation in bulk properties from those reported for purified glycerol, while the surface tension shows only minor deviations from literature values. Overall, (bio)glycerol mixes ideally with water, suggesting that marginal changes in the local structure of the solvent medium occur. This makes these mixtures suitable media for solubilizing surfactants with little effect on hydrophobic interactions, while adding all the benefits of glycerol (e.g., viscosity regulation, prevention of freezing and evaporation).

To support this hypothesis in the context of environmentally friendly formulation design, in the second part of this study, the aggregation properties of rhamnolipids in water–bioglycerol mixtures were investigated by surface tension and self‐diffusion measurements. Rhamnolipid aggregation is hindered by bioglycerol only at *w*_BG_ higher than 40%–50%, with a significant increase in critical micelle concentration observed only at *w*_BG_ = 80%. Interestingly, the magnitude of these variations suggests a behavior similar to that observed for conventional anionic surfactants, which could be interpreted in terms of the slightly reduced dielectric constant that enhances the electrostatic repulsion between the negatively charged headgroups. The same effect justifies the decreasing dimension of the aggregates as estimated from the self‐diffusion coefficients measured by NMR DOSY experiments.

On the other hand, surface adsorption at the water–air interface is hardly affected by bioglycerol over the whole concentration range, proving that rhamnolipids are more effective at stabilizing the surface tension of complex mixtures than conventional synthetic ionic surfactants. Overall, these results encourage the exploration of rhamnolipids as components of products characterized by a high concentration of glycerol as cosolvent and prompt the valorization of bioglycerol as a key component of these formulations.

## 5 Experimental Section

5.1

5.1.1

##### Materials

The sample of rhamnolipids (named Rha) used for our study was a biotechnological product obtained by *P. aeruginosa* culture using vegetable oils as the carbon source and was purchased from AGAE Technologies (Corvallis, OR, USA). It is a heterogeneous mixture containing 90% by mass of rhamnolipids, of which 60% were mono‐rhamnolipids and 40% were di‐rhamnolipids; 20% were single‐tailed and 80% were double‐tailed species; 70% were saturated and 30% were mono‐unsaturated fatty acids. The tail lengths ranged from C6 to C16, with a dominant presence of C10.^[^ ^59^, ^72^ ^]^

Soybean oil was purchased in a local food store, and methanol (purity ≥99.8%) was acquired from VWR International and KOH (≥85%) from Sigma Aldrich. For the glycerol purification process, acetic acid (≥99.8%) was purchased from VWR International, Amberlite 20 °C, and activated carbon were supplied by Sigma Aldrich, whereas Amberlite IRN‐78 was sourced from Supelco.

Sodium phosphate mono‐ and dihydrate (purity > 99%), used to prepare the buffer aqueous solutions, was purchased from Sigma‐Aldrich (Milan, Italy). As a solvent for all sample preparation, we used ultrapure deionized water from a Millipore Milli‐Q system, with the only exception of samples for NMR‐DOSY experiments that were prepared using D_2_O (99.8 atom % D, obtained from Merck, Milan, Italy). All the samples were filtered through a 0.22 μm filter.

##### Glycerol Production and Purification

Crude glycerol was obtained through the transesterification of triglycerides with methanol, promoted by KOH. The reaction was conducted in a batch glassy vessel at reflux condition, loading soybean oil/methanol 1:6 molar ratio, 1 wt% KOH. The reaction time was set to 1 h.

The purification process of crude glycerol involved several key steps. First, neutralization with acetic acid was performed to remove excess potassium hydroxide used as a catalyst in the transesterification reaction, reaching a neutral pH. This was followed by filtration to eliminate salts, such as potassium acetate, produced during neutralization. Methanol was removed through vacuum evaporation, as an excess was typically used in the transesterification process. The crude glycerol was then treated with ion‐exchange resins to remove residual inorganic salts and free ion impurities. This was achieved by pumping the crude glycerol through columns packed with Amberlite IRN‐78 and Amberlite 20 °C, which adsorbed free anions and cations. Finally, an adsorption step with activated carbon was performed to decolorize and further purify glycerol, resulting in a clear final product. High‐performance liquid chromatography (HPLC) analysis was made to evaluate the final purity, which was equal to 99.5%. The rest consisted of residual ash content, which was the potassium acetate, determined according to the ISO 2098‐1972 Standard method.

##### Refractive Index, Density, and Viscosity of Bioglycerol Aqueous Mixtures

The refractive index, density, and viscosity of mixture of bioglycerol and 4 mM sodium phosphate‐buffered aqueous solutions at pH 7.1 were assessed at a temperature of 25 °C across a broad spectrum of bioglycerol weight percent (*w*_BG_), ranging from 0 to 80%.

The refractive index was measured using an Abbe refractometer NAR‐3 T (Atago, Tokyo, Japan).^[^ ^103^ ^]^ Density measurements were performed with a densimeter DMA 5000 (Anton Paar, Graz, Austria).^[^ ^104^ ^]^ Prior to each measurement, the densimeter's capillary was cleansed with hydrochloric acid, and the device was calibrated with double‐distilled water and dry air.

The kinematic viscosity of the bioglycerol mixtures was determined using an Ubbelohde viscometer, with a water flow time of 110 s (Cannon, State College, PA, USA).^[^ ^105^ ^]^ Refractive index and density were measured on samples that were prepared independently, while viscosity was determined by gradually diluting concentrated bioglycerol (*w*_BG_ = 80%) in the viscometer with precisely weighed amounts of the buffered aqueous solution.

##### Surface Tension of Rha‐Bioglycerol Aqueous Mixtures

The surface tension, *γ*, of Rha aqueous solutions buffered at a pH of 7.1 with 4 mM sodium phosphate was measured in the presence of varying concentrations of bioglycerol, specifically at *w*_BG_ = 5, 10, 15, 20, 30, 40, 50, 60, 70, and 80%. These measurements were conducted at a constant temperature of 25 °C utilizing a Sigma 70 tensiometer (KSV, Stockholm, Sweden) and the Du Noüy ring technique.^[^ ^106^ ^]^ Moreover, the surface tension of bioglycerol aqueous mixtures devoid of rhamnolipids was also determined. The rising velocity of the platinum ring was checked to be low enough to allow the surfactant adsorption at the air/liquid interface to reach equilibrium even in highly viscous mixtures.^[^ ^107^ ^]^ Successive aliquots of a concentrated buffered Rha solution, containing a predetermined amount of bioglycerol, were poured into a vessel containing a known volume of the buffered solution with an identical bioglycerol content. Following the addition of each aliquot, the mixture was agitated using a magnetic stirrer and allowed to equilibrate for 5 min before measuring the surface tension. At the conclusion of each titration experiment, the pH of the final mixture was verified to ensure it remained within 0.3 units of the initial pH value.

We also calculated the minimum area occupied by a surfactant molecule at the air/solution interface at saturation, *A*_min_, by means of the Gibbs adsorption isotherm equation.

$$A_{\min}=-\frac{nRT}{{\text{N}}_{A}}\frac{1}{\frac{d\gamma }{dlnc}}$$

where *N*_A_ is the Avogadro constant*, R* is the gas constant, *T* is the absolute temperature, d*γ*/d ln *c* is the slope of the plot of *γ* versus the logarithm of surfactant concentration *c* in the premicellar region, and *n* is a coefficient that takes into account the dissociation of ionic surfactants, known as the Gibbs prefactor. In this study, a value of 2 was used because of the dissociation of the rhamnolipid carboxylic group at pH = 7.1 > pK_a_ = 5.5.^[^ ^52^ ^]^ Since the dissociation of carboxylic acid in aqueous mixtures has been shown to be only slightly affected by the presence of glycerol, even in large amounts,^[^ ^108^, ^109^ ^]^ it is reasonable to assume that Rha molecules retain the negative charge in all systems studied in this work.

##### NMR DOSY of Rha‐Bioglycerol Aqueous Mixtures

Samples for NMR analysis were prepared in D_2_O at fixed Rha concentration (13.3 mg ml^−1^) and increasing bioglycerol content (i.e., w_BG_ = 10%, 30%, 50%, 70% and with no bioglycerol for comparison), then they were transferred into borosilicate tubes with a diameter of 5 mm and a length of 17.8 cm. The 1 H 1D and pseudo 2D diffusion ordered spectroscopy (DOSY) experiments were performed at 25 °C on a Bruker Avance spectrometer with a proton resonance frequency of 400 MHz and equipped with an iProbe (Bruker, Rhenistetten‐Forchheim, Germany). The DOSY experiment allows the measurement of the self‐diffusion values of each component *i* of the mixture, *D*_*i*_, provided that it is responsible for a signal in the monodimensional spectra during which a pulsed field gradient of varying strength is applied. The pseudo 2D DOSY spectra were obtained using a stimulated echo pulse sequence with bipolar gradients (*stebpgp1s*), which involved two fundamental delays: the little delta (*δ*), which determines the pulse length, and the big delta (*Δ*), which represents the diffusion time of the molecules. The decays of the signals, differently affected by the strength of the gradient, were analyzed according to the Stejskal–Tanner equation.

$$I_{G}={I_{0}e}^{\left[-{\left(\gamma \delta G\right)}^{2}D_{i}\left(\Delta -\frac{\delta }{3}\right)\right]}$$

where *I*_*G*_ is the signal intensity at a given gradient strength *G*, *I*_0_ is the signal intensity in the absence of applied gradient, and *γ* is the gyromagnetic constant. Measurements to calibrate the *G* value were performed on heavy water with traces of light water as a reference sample, whose self‐diffusion coefficient is *D*_HDO_ = 1.872 × 10^−9^ m^2^ s^−1^.^[^ ^110^ ^]^ For the acquisition, 19,180 free induction decay points, 16 scans, a spectral window of 12 ppm, a *d* of 4 ms, and a *D* of 60 ms were set. Totally, 16 spectra were acquired using a linear gradient strength ramp from 2 to 98%. The spectra were processed and analyzed using the Dynamic Center software included in TopSpin 4.4.0 (Bruker) following the signal intensities of the methylene and terminal methyl groups of the acyl chains (chemical shift = 1.2 and 0.8 ppm, respectively). The bioglycerol self‐diffusion coefficient was measured from the signals at 3.3–3.5 ppm. The water self‐diffusion coefficient was determined following the signal intensities of the OH groups (chemical shift = 4.7 ppm). In fact, due to the proton exchange between the OH groups of rhamnolipid and bioglycerol and those of heavy water, this signal was due to all three species. Since the exchange is much faster than the stimulated spin‐echo sequence, a single self‐diffusion coefficient, *D*_OH_, was determined, which is an average value according to the relationship:

$$D_{\mathrm{OH}}=\frac{\left(2n_{w}D_{w}+3n_{\mathrm{BG}}D_{\mathrm{BG}}+5n_{\mathrm{Rha}}D_{\mathrm{Rha}}\right)}{\left(2n_{w}+3n_{\mathrm{BG}}+5n_{\mathrm{Rha}}\right)}$$

where *n*_i_ is the number of moles of each species in the sample and the denominator represents the total number of hydroxyl protons. Since *D*_BG_ and *D*_Rha_ can be determined independently, this relationship allows the calculation of *D*_w_.

To correct the self‐diffusion coefficients obtained in deuterated aqueous solutions back to those in normal water, we multiplied them by a factor of 1.23,^[^ ^110^, ^111^ ^]^ which is the ratio of the intradiffusion coefficients of normal and deuterated water. This correction was verified to hold in glycerol–aqueous mixtures up to high glycerol contents.^[^ ^108^ ^]^

##### Data Representation and Fitting

Refractive index, density, viscosity, and surface tension of water–bioglycerol binary systems were plotted as a function of *w*_BG_, and experimental data were fitted to established models using Origin 2018 (OriginLab Corporation, Northampton, MA, USA). In all the cases, the mean values derived from three independent measurements were plotted, with standard deviations to represent uncertainties, and the quality of the fit was evaluated on the basis of the *R*‐squared value, i.e., the coefficient of determination.

Surface tension profiles and surface and aggregation properties of rhamnolipids as a function of the bioglycerol content were represented and analyzed in the same way. Critical micelle concentration (*cmc*) values were obtained from tensiometric titration plots, in which the surface tension was reported as a function of the Rha content; in particular, they were identified as the intersection points between the fitted lines in the concentration range where surface tension decreased with increasing Rha concentration (premicellar region) and the range where surface tension remained relatively stable despite rising Rha concentrations (micellar region). This method introduced a maximum uncertainty of 10% in the calculated *cmc* values that were reported in the corresponding plot as error bar. The slope of the line fitting the surface tension data in the premicellar region was utilized to determine *A*_min_.

Plots of self‐diffusion coefficients of water, bioglycerol, and rhamnolipid and of hydrodynamic radii of Rha aggregates as a function of the bioglycerol content were all prepared using Origin 2018.

## Conflict of Interest

The authors declare no conflict of interest.

## Supporting information

Supplementary Material

## Data Availability

The data that support the findings of this study are available from the corresponding author upon reasonable request.
