# Supplementary material for: Rhamnolipid Self‐Aggregation in Water–Bioglycerol Mixtures: Byproduct Valorization for Sustainable Formulation Design
Source: Chempluschem. 2025 Jun 17;90(9):e202500163. doi: 10.1002/cplu.202500163 (PMC12435146; doi:10.1002/cplu.202500163)
Supplement: Supplementary file 1 — Supplementary Material [file CPLU-90-e202500163-s001.pdf]

Supplementary Information

For the Article

Rhamnolipid Self-aggregation in Water-Bioglycerol Mixtures: By-product Valorization for Sustainable Formulation Design

Rodolfo Esposito[1,2,*], Matilde Tancredi[1,2,*], Michela Buonocore[1], Carlo Carandente Coscia[1,2], Francesco Taddeo[1], Vincenzo Russo[1], Delia Picone[1], Gerardino D'Errico[1,2, †] and Irene Russo Krauss [1,2,†]

[1]Department of Chemical Sciences, University of Naples Federico II, Via Cintia 4, Complesso Universitario di Monte Sant'Angelo, I-80126 Naples, Italy;

[2]Consorzio Interuniversitario per lo Sviluppo dei Sistemi a Grande Interfase (CSGI), Via della Lastruccia 3, I-50019 Florence, Italy

[†]Correspondence: irene.russokrauss@unina.it (I.R.K.); Tel.: +39-081-674274. gerardino.derrico@unina.it (G.D'E); Tel.: +39-081-674227

[*]These authors equally contributed to this work

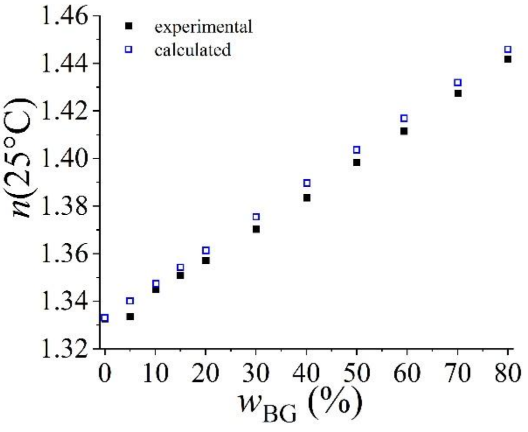

a)

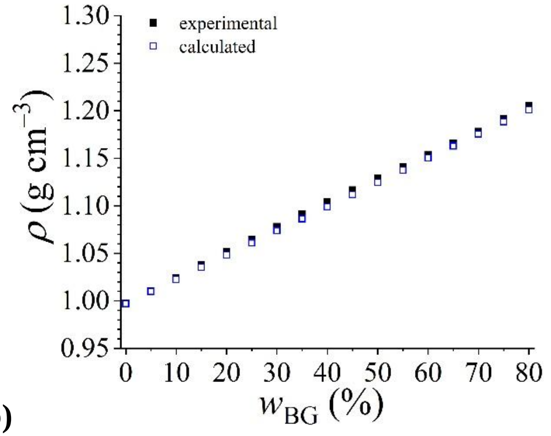

b)

Figure S1 Comparison between experimental (filled squares) and calculated (empty squares) values of refractive index **(a)** and density **(b)** of water-bioglycerol mixtures as a function of bioglycerol content

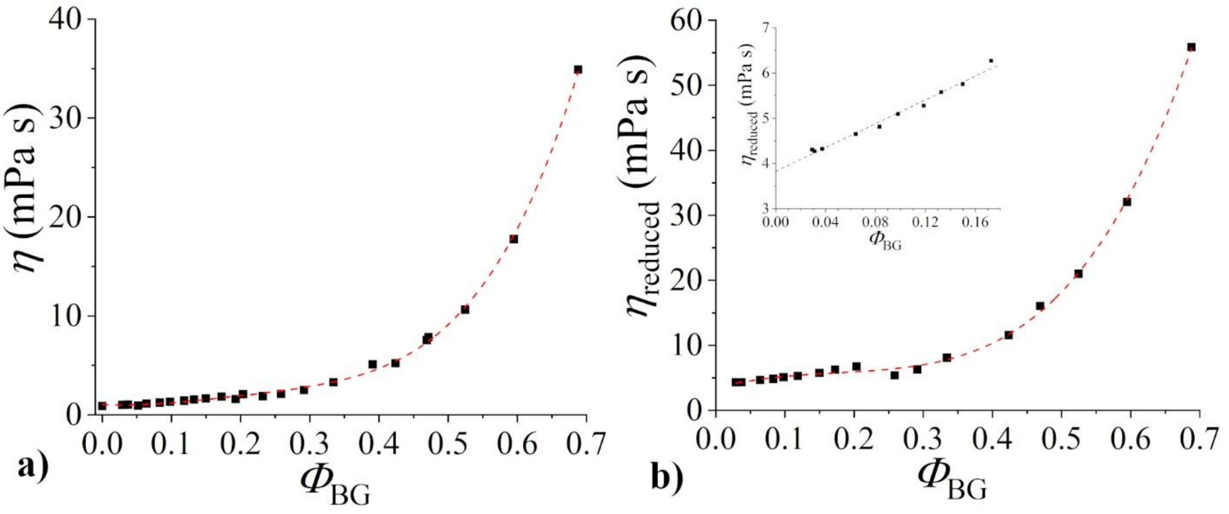

Figure S2 Viscosity **(a)** and reduced viscosity **(b)** of bioglycerol mixtures in 4 mM sodium phosphate buffer solution at pH 7.1, reported as a function of bioglycerol volume fraction Φ_{BG}. Red dashed lines are guide to the eye. In the inset of panel b) the fitting of reduced viscosity at low Φ_{BG} for determination of the the intrinsic viscosity is shown. An intrinsic viscosity of 3.82 mPa s was determined.

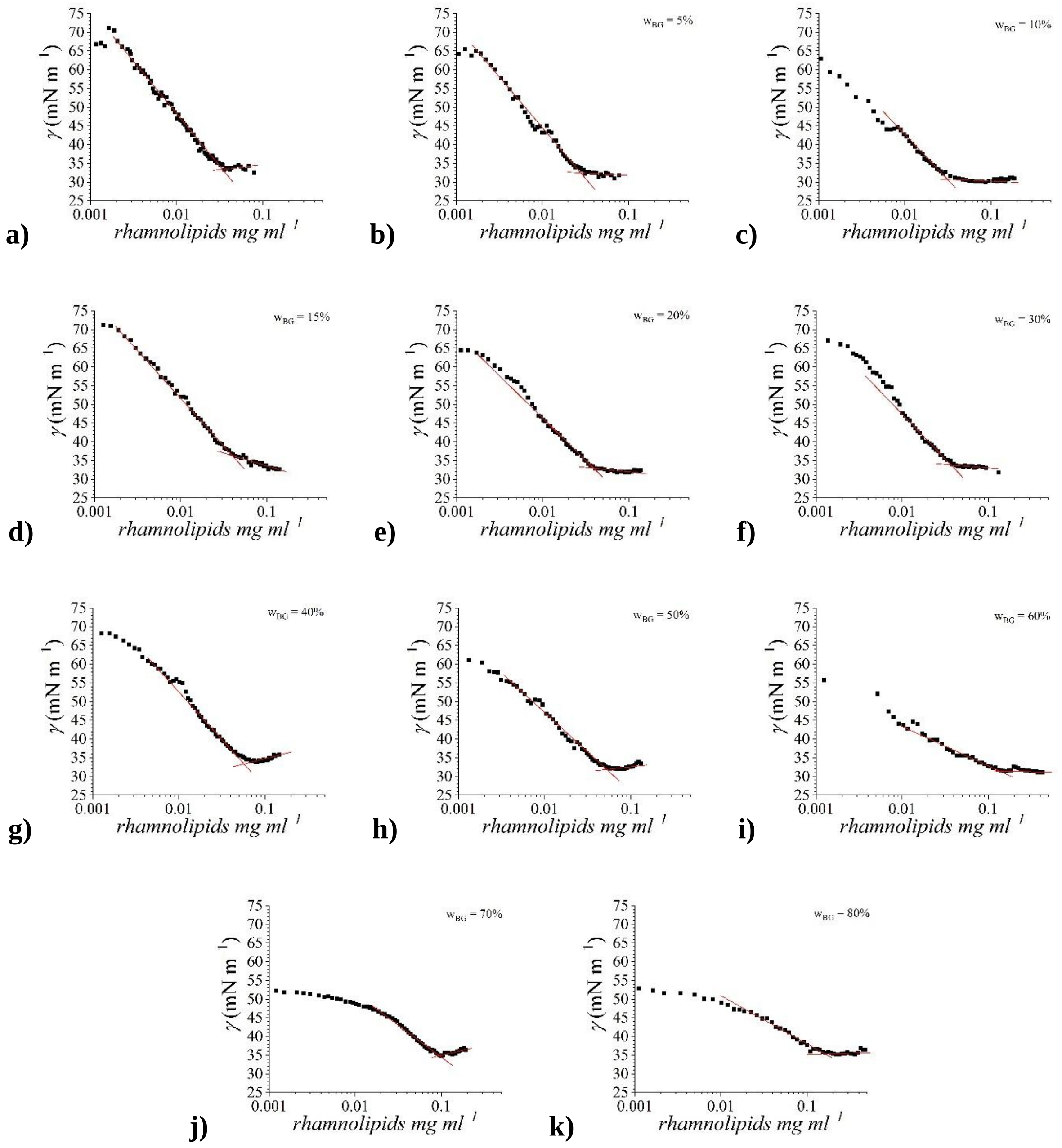

Figure S3. Tensiometric titration curves of rhamnolipids in 4 mM sodium phosphate buffer solution at pH 7.1 in the presence of increasing contents of bioglycerol: (**a**) no bioglycerol, (**b**) w_{BG} = 5%, (**c**) w_{BG} = 10%, (**d**) w_{BG} = 15%, (**e**) w_{BG} = 20%, (**f**) w_{BG} = 30%, (**g**) w_{BG}= 40%, (**h**) w_{BG} = 50%, (**i**) w_{BG} = 60%, (**j**) w_{BG} = 70%, (**k**) w_{BG} = 80%. Experimental data are represented by black squares, while red lines represent the best fitting of data in the pre-micellar and post-micellar regions, in this way inflection points corresponding to *cmc* values are explicitly shown.

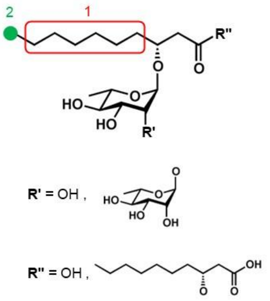

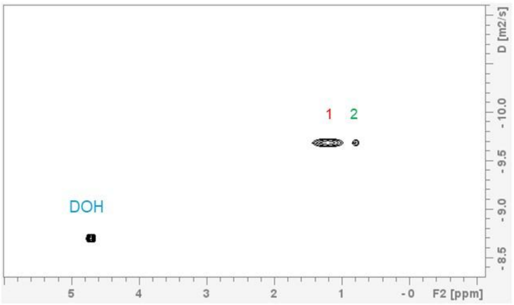

Signal	δ (ppm)	D (m^2/s)	Error
DOH	4.78	1.72e^{-9}	7.63e^{-13}
1	1.28	1.79e^{-10}	1.39e^{-12}
2	0.87	1.82e^{-10}	3.99e^{-12}

a)

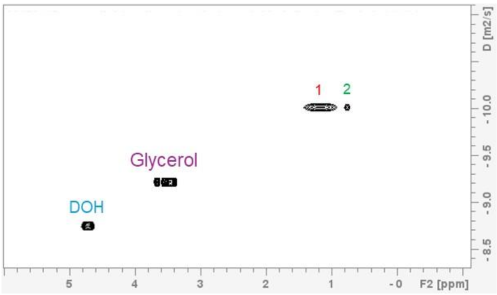

Signal	δ (ppm)	D (m^2/s)	Error
DOH	4.82	1.50e^{-9}	1.43e^{-12}
Glycerol	3.55-3.78	5.33e^{-10}	6.95e^{-14}
1	1.29	8.71e^{-11}	2.56e^{-13}
2	0.88	8.05e^{-11}	7.07e^{-13}

b)

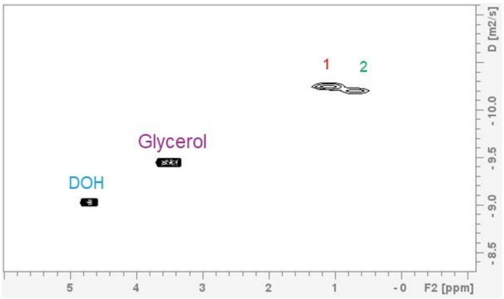

Signal	δ (ppm)	D (m^2/s)	Error
DOH	4.88	7.98e^{-10}	1.27e^{-14}
Glycerol	3.54-3.76	3.04e^{-10}	6.78e^{-15}
1	1.28	5.10e^{-11}	1.82e^{-13}
2	0.87	5.60e^{-11}	3.51e^{-13}

c)

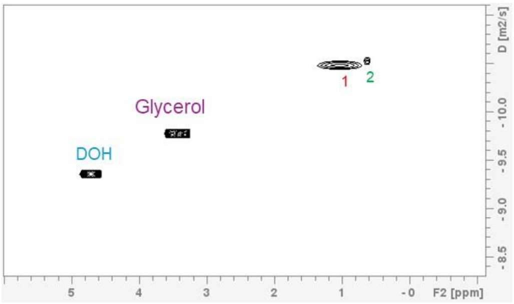

Signal	δ (ppm)	D (m^2/s)	Error
DOH	4.94	3.98e^{-10}	4.64e^{-15}
Glycerol	3.53-3.75	1.47e^{-10}	1.805e^{-15}
1	1.27	2.80e^{-11}	1.90e^{-13}
2	0.86	2.47e^{-11}	3.66e^{-13}

d)

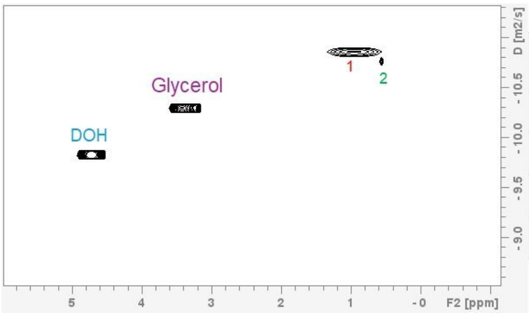

Signal	δ (ppm)	D (m^2/s)	Error
DOH	5.02	1.24e^{-10}	1.06e^{-15}
Glycerol	3.52-3.73	4.33e^{-11}	7.05e^{-16}
1	1.26	1.22e^{-11}	1.75e^{-13}
2	0.87	1.40e^{-11}	4.11e^{-13}

e)

Figure S4 Pseudo 2D DOSY spectra correlating the chemical shifts (F2, ppm) with the diffusion coefficients (D, m²/s) of Rha in D_2O and in the presence of increasing contents of bioglycerol - a) no bioglycerol, b) w_{BG}=10%, c) w_{BG}=30%, d) w_{BG}=50%, and e) w_{BG}=70% - and table reporting the obtained diffusion values for each species. The peaks observed for Rha are attributed to the methyl and methylene groups in the lipid chains, as evidenced in the molecular structure on the left of panel a.
